# THE ARCH NETWORK: LOOKING AHEAD

**DOI:** 10.1093/geroni/igae098.1549

**Published:** 2024-12-31

**Authors:** Fernando Murillo

**Affiliations:** Humane Prison Hospice Project, San Francisco, California, United States

## Abstract

It is clear that the commitment to addressing the pressing public health problem of mass incarceration and aging is growing. With every ARCH Network meeting, there are more of us who care. We even started a GSA Incarceration and Aging Interest Group! This presentation provides the perspective of an individual with lived experience as a pastoral care services worker for terminally ill incarcerated persons. Perspectives such as these are an essential to the mission of the ARCH Network. Looking ahead, we will continue to build new collaborations and partnerships and strengthen those that already exist. We will continue to refine our research agenda. We will continue to disseminate our work to make a difference in the lives of those impacted by the intersection of incarceration and aging. The ARCH Network has much to be proud of, yet we know that there is still even more that we can do.

